# Classical epithelial-mesenchymal transition (EMT) and alternative cell death process-driven blebbishield metastatic-witch (BMW) pathways to cancer metastasis

**DOI:** 10.1038/s41392-022-01132-6

**Published:** 2022-08-23

**Authors:** Goodwin G. Jinesh, Andrew S. Brohl

**Affiliations:** 1 Department of Molecular Oncology, 12902 USF Magnolia Drive, H. Lee Moffitt Cancer Center & Research Institute, Tampa, 33612 FL USA; 2 Sarcoma Department, 12902 USF Magnolia Drive, H. Lee Moffitt Cancer Center & Research Institute, Tampa, 33612 FL USA

**Keywords:** Cancer stem cells, Cell biology, Metastasis, Tumour immunology, Cancer microenvironment

## Abstract

Metastasis is a pivotal event that accelerates the prognosis of cancer patients towards mortality. Therapies that aim to induce cell death in metastatic cells require a more detailed understanding of the metastasis for better mitigation. Towards this goal, we discuss the details of two distinct but overlapping pathways of metastasis: a classical reversible epithelial-to-mesenchymal transition (hybrid-EMT)-driven transport pathway and an alternative cell death process-driven blebbishield metastatic-witch (BMW) transport pathway involving reversible cell death process. The knowledge about the EMT and BMW pathways is important for the therapy of metastatic cancers as these pathways confer drug resistance coupled to immune evasion/suppression. We initially discuss the EMT pathway and compare it with the BMW pathway in the contexts of coordinated oncogenic, metabolic, immunologic, and cell biological events that drive metastasis. In particular, we discuss how the cell death environment involving apoptosis, ferroptosis, necroptosis, and NETosis in BMW or EMT pathways recruits immune cells, fuses with it, migrates, permeabilizes vasculature, and settles at distant sites to establish metastasis. Finally, we discuss the therapeutic targets that are common to both EMT and BMW pathways.

## Introduction to metastasis: the devil in its essential details

Primary benign solid tumors in various tissue types remain to grow in the same location where the neoplastic cells were initially formed. Some of the benign primary tumors may remain in a dormant state without considerable growth for a long time but are being constantly exposed to microenvironmental and immune selection pressures. During this period, the tumor cells learn to divide at a higher frequency and manage to survive immune, therapeutic (if subjected to), and microenvironmental insults. The most pivotal event that accelerates the prognosis of cancer patients towards mortality is metastasis,^1–4^ where the cancer cells learn to migrate, invade and settle down at preferred sites outside primary tumors and continue to grow.^2^ The metastatic migration can be of long distance through blood circulation (hematogenous metastasis^5^), through lymphatics (generally through local draining lymph nodes^6^), or of short distance (next to the primary tumor as field-effect^7^). Epithelial-to-mesenchymal transition (EMT) of tumor cells has been implicated in the process of metastatic migration for decades.^8^ Metastatic spread has a predominant preference to lymph nodes,^9^ liver,^10,11^ lungs,^11^ skin,^12^ brain,^13^ or bone^14^ with an exhibition of acquired therapy resistance.^15^ Chemo/radiotherapies (in patients) and preclinical therapeutics (in vitro/animal models) educate the cancer stem cells (CSCs) to withstand therapy (despite various forms of the cell death process is initiated).^16–20^ This process is coupled to cellular transformation,^21^ which selects the CSCs that exhibit enhanced glycolysis,^19^ a consistent characteristic feature of metastatic cancer cells.^22^ These facts indicate the point that, an apoptosis/cell death process-dependent cellular transformation is also involved in metastasis^23,24^ in addition to the well-worked-out classical epithelial-to-mesenchymal transition (EMT) mode of metastasis.^25^

**Fig. 1:**
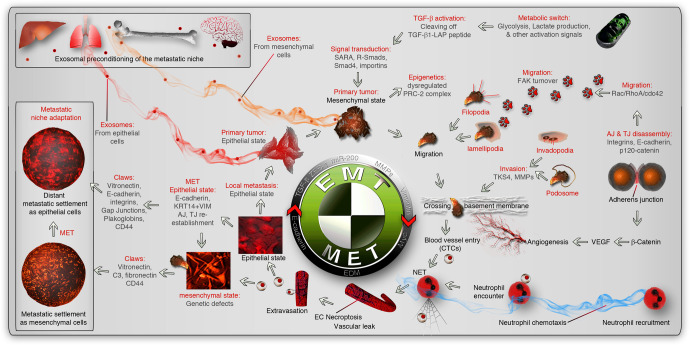
Classical EMT pathway for metastasis. The metastatic events start with the primary tumor producing exosome-mediated or alternative preconditioning of the metastatic path and niche (indicated by red/orange smoky trail). The metastatic environment is indicated by inset boxes. Altered glycolysis in primary tumor cells or other ECM remodeling triggers TGF-β activation by processing the latency-associate peptide (LAP) to regulate transcription by activation of receptor-regulated Smads-2/3 (R-Smads) through Smad anchor for receptor activation (SARA). Resultant EMT-inducing transcription factors induce a mesenchymal transition from epithelial phenotype while suppressing apoptosis through dysregulated polycomb repressor complex-2 (PRC2). Mesenchymal phenotype is associated with adherens junction (AJ) and tight junction (TJ) disassembly to promote migration (through FAK turnover, lamellipodia, and filopodia) and invasion (through invadopodia and podosomes to cross basement membranes) phenotypes to reach the circulation or through promoting local angiogenesis by VEGF. Inside the blood vessel, neutrophils are attracted by chemotaxis (blue smoky trail), which promotes neutrophil extracellular trap (NET) and necroptosis to facilitate vascular exit. This exposes the circulating tumor cells (CTCs) to a new microenvironment which may or may not promote the reversal of EMT (MET) through the promotion of the epithelial differentiation module (EDM). The extravasated cells use different adhesion cues (claws) to settle and adapt to the new metastatic niche. The reversibility of EMT may support another cycle of EMT and MET

**Fig. 2:**
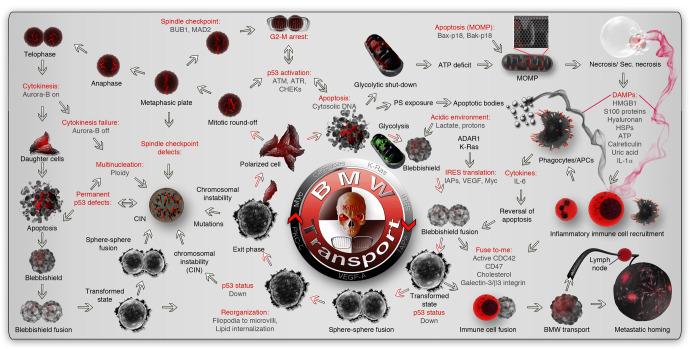
Alternative cell death process-dependent BMW pathway for metastasis. The metastatic events start with the initiation of the reversible cell death process. Upon initiation of apoptosis, an acidic environment, and enhanced endocytosis initiate the cell fusion behavior in apoptotic cells. ARAR1 and K-Ras drive the protection of anti-apoptotic mRNAs (IAPs) and IRES translation. Membrane fusion initiates the reassembly of apoptotic blebs to form blebbishields. Blebbishield-blebbishield fusion results in the transformed spheroid state with multiple copies of genomes inside. The nuclear membrane disappears during this stage. Depending on mitochondrial dysfunction and ATP scarcity, the cells can enter an irreversible secondary necrotic state. In parallel, DAMPs (indicated by black or maroon smoky trails from apoptotic and secondary necrotic cells, respectively) attract immune cells to the scene promoting an immune cell-cancer cell hybrid (analogous to a transformed state). The hybrids acquire migratory capacity and use homing signals of immune cells to reach target organs (BMW transport) and use immune cell identity to evade the immune system to establish metastasis. During the transformed spheroid state, p53 is suppressed from expression to suppress apoptosis. The transformed spheres then reorganize their fusogenic lipid membranes (retracted inside from the surface) and reform the nuclear membrane and release individual polarized cancer cells outside the spheroid (Exit phase). The cells released from the transformed state can be polyploid, aneuploid, or diploid to augment genomic instability and heterogeneity. The exited cells may undergo further round of apoptosis due to reactivation of genomic checkpoints, undergo cell division, or undergo cell cycle arrest to augment genomic instability. DAMP damage-associated molecular patterns, MOMP mitochondrial outer membrane permeabilization, BMW transport blebbishield-mediated metastatic-witch transport, PS phosphatidyl serine, CIN chromosomal instability, HSPs heat-shock proteins

In this review, we classify the sequence of events leading to metastasis into two main pathways, viz., classical reversible EMT (Fig. 1) and alternative cell death process-driven blebbishield metastatic-witch (BMW) (Fig. 2) transport pathways. A huge body of transcription-based evidences point to the fact that, genetic regulation (coding and non-coding RNAs and epigenetic mechanisms) of metastasis is primarily executed through EMT and evasion of cell death (comprehensively reviewed in ref. ^24^), supporting the need for EMT versus BMW classification to better understand and treat the sub-types of metastasis. We introduce the core aspects of the classical EMT pathway of metastasis first,^1,8^ and then describe the emerging alternate BMW metastasis pathway in detail. We add special notes on how the process of cell death initiates cellular transformation, attracts immune cells, tackles immune cells, migrates, extravasates, and establishes distant metastasis. In addition, we point out the similarities and key discriminatory characteristics of EMT and BMW pathways. Finally, we focus on therapeutic targeting of metastasis by giving an outline of targets that are shared by both EMT and BMW pathways.

## Reversible hybrid-EMT, the slow-and-steady pathway for metastasis

**Fig. 3:**
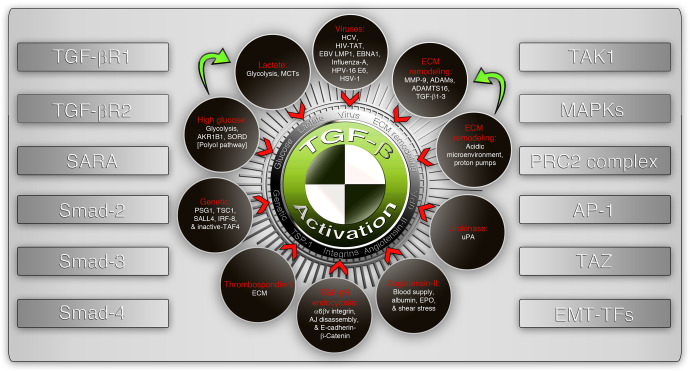
Activators and signaling components of the TGF-β signaling pathway. A non-exhaustive list of activators of TGF-β signaling (shown in dark brown circles) where one activator may lead to the convergence with the other (green arrows). The downstream signaling receptors and signal transducers (left panel) and important downstream co-operative molecules regulating epithelial-mesenchymal transition (right panel) are listed as side panels. SARA Smad anchor for receptor activation, TAK1 TGF-β activated kinase-1, MAPKs mitogen-activated protein kinases, PRC2 polycomb-repressive complex-2, AP-1 activator protein-1 (Jun/Fos/Fra), EMT-TFs epithelial-to-mesenchymal transition regulatory transcription factors, MCTs monocarboxylate transporters, ECM extracellular matrix, EPO erythropoietin, AJ adherens junction

TGF-β signaling acts as a prominent regulator of the EMT process^8^ in which, the rigid epithelial cells (interlocked with neighboring cells) acquire a flexible morphological phenotype. TGF-β signaling can be activated by a plethora of signals, including but not limited to TGF-β isoforms-1-3, acidic microenvironment (through proton pumps or through metabolic lactate accumulation outside the cells through monocarboxylate transporters: MCTs),^26^ urokinase,^27^ shear stress,^28^ thrombospondin-1 (TSP1/*THBS1*),^26^ high glucose,^29^ multiple viruses^30^, and integrin-cadherin enriched adherens junction (AJ) remodeling (Figs. 1, 3). Most of these activators result in the processing of the latent-TGF-β binding peptide in the TGF-β ligand,^31^ which stimulates its maturation and binding to its receptors in a paracrine, autocrine, or endocrine fashion. TGF-β receptor activation results in the phosphorylation of R-Smad-2, 3 through Smad anchor and receptor activation (SARA).^32^ Phosphorylated R-Smads then interact with Smad4 to transduce signals to the nucleus for transcriptional regulation.^8,32^ Smads promote EMT-transcription factors (TFs: Snail, Twist1, Zeb1, and others) to orchestrate the EMT process through transcription.^8^ TGF-β signaling is very essential for normal biological processes such as wound healing^8^ and apoptosis^33,34^ however, TGF-β-driven transcription is largely remodeled by dysregulated PRC2 complex-mediated epigenetics^35^ in the metastatic context.^36,37^ TGF-β can also signal through TAK1,^38^ Ras/Raf/MAPK/ERKs,^39^ p38-MAPK,^40,41^ JNK/AP-1,^42^ TAZ,^43^ which could complement the EMT-driven metastasis process by suppressing apoptosis^42^ (Fig. 3).

The classic understanding that epithelial-to-mesenchymal transition is essential for metastasis,^1^ remained a prominent mode of metastatic migration for a long time.^24^ However, the requirement of EMT for metastasis is refuted.^44^ Recent understanding of EMT in the context of metastasis has revealed that a reversible hybrid-EMT phenotype (EMT followed by mesenchymal-to-epithelial transition: by cells that co-express keratin-14 [*KRT14*] and vimentin [*VIM*]) contributes to metastasis^25,45–51^ (Fig. 1), whereas pure EMT (non-reversible) contributes to drug resistance^38,44,52^ and not metastasis.^44,53^ Notably, there are reports of mesenchymal metastatic tumors^54,55^ indicating the fact that the reversal of EMT may not be a strict requirement. Epithelial cells usually form rigid zipper-like cell-to-cell adhesion (adherens junction: AJ) with adjacent cells through molecules such as E-cadherin, p120 catenin, integrins, and other cell surface molecules.^56,57^ The cell-to-cell contacts are further reinforced with tight junctions (TJ) and desmosomes while permitting cell-to-cell communication through gap junctions.^8^ This cell adhesion property does not support migration in vivo despite epithelial cells can migrate in vitro after trypsinization. Transforming growth factor-β (TGF-β), activation (Figs. 1, 3), and or viral particles stimulate remodeling of the cell surface adherens junction (AJ) and tight junction (TJ) molecules^45^ through p120 catenin.^58,59^ AJ loss and TJ loss activate a CAMK2–CD44–SRC axis that promotes YAP1 nuclear translocation and ZEB1 expression that stimulates the mesenchymal state through transcription.^45^ Interestingly, AJ and TJ disassembly, CAMK2-CD44-SRC axis, and ZEB1 activation cascade is triggered by the loss of function of the *FAT1* gene.^45^ Despite these events are downstream of *FAT1* gene, the loss of contacts with adjacent cells is classically marked by RhoA GTPase activation.^60^

The mesenchymal state aids migration and invasion through multiple mechanisms (including epithelial cell migratory mechanisms), including but not limited to the formation of lamellipodia,^61,62^ filopodia,^63^ pseudopodia,^64^ and focal adhesion kinase-mediated focal adhesion turnover^65,66^ (Fig. 1). This step is largely controlled by small GTPase activations as Rac1 regulates lamellipodia,^67^ cdc42 regulates filopodia^68,69^ and RhoA regulates AJ disassembly.^70^ Activation of these three small GTPases is tightly associated with focal adhesion turnover by focal adhesion kinase (FAK) to execute footsteps of migration^71^ (Fig. 1). TGF-β super-regulates FAK, RhoA, Rac, and cdc42 signaling to remodel the actin polymerization pattern to promote migratory features.^72,73^ Despite the flexible mesenchymal state allows migration, the cells need invasive potential to cross basement membranes,^74–76^ a major barrier to metastasis (Fig. 1). The invasion process is largely controlled by extracellular matrix (ECM) by matrix-metalloproteinases (MMPs) and cytoskeleton remodeling by TKS4 (Tyrosine kinase substrate with four SH3 domains) which are enriched or hyperactivated in the cellular structures such as podosomes and invadopodia.^77,78^ Podosomes and invadopodia digest the local basement membranes through MMP action^78,79^ (Fig. 1).

AJ disassembly is tightly associated with β-catenin activation^80^ as β-catenin is anchored at the interior side of the plasma membrane by E-cadherin and 4.1 R, an AJ-to-cytoskeleton linking component.^81^ The transcriptional activity of β-catenin promotes VEGF signaling,^82^ which in concert with already active TGF-β signaling to promote permeability and entry through local blood vessels or promote neovascularization through angiogenesis/neovascularization and then enter the circulation^5^ (Fig. 1). Once in the circulation, the bloodstream can passively carry the mesenchymal tumor cells (as circulating tumor cells: CTCs) to various organs, where the microenvironmental and CTC cellular cues determine where the mesenchymal cells should extravasate. One such major cue is neutrophil interaction and neutrophil extracellular trap (NET) formation by a peculiar cell death process, NETosis^83^ (Fig. 1). Presumably, NETosis primarily helps to sequester the mesenchymal cancer cells at the extravasation site, but tumor cell-induced TGF-β-activated kinase-1 (TAK1)-driven^84^ necroptosis of endothelial cells (another form of cell death) which helps the cancer cells to extravasate in a violent mode^85^ compared to diapedesis mode of extravasation (Fig. 1).

Upon reaching the metastatic target site, the cells reverse EMT (undergo MET) to acquire an epithelial phenotype to colonize as metastatic cells. This process of MET change at a metastatic site may require an additional one or two cell divisions^86^ suggesting a niche adaptation procedure involvement. This niche adaptation potentially involves the suppression of the TGF-β signaling target and EMT-inducer Prrx1.^53^ The MET change is driven by acquiring Rac1 activation and potentially ceasing RhoA activity to re-establish AJ and other cell-cell adhesion mechanisms.^87^ The metastatic cells can activate an array of adhesive mechanisms (claws to cling on to the target site) to settle at metastatic sites primarily through cell adhesion molecules such as CD44, vitronectin, E-cadherin, integrins, plakoglobins, and so on (Fig. 1). For example, CD44 regulates adhesion through Rac1 activation.^67,88^

The metastatic foci, in general, have enhanced glycolytic capacity.^89^ This reflects the underlying cellular signaling context of metastatic cells. For example, high AJ turnover results in the activation of a c-Src oncogene,^45^ which ignites glycolysis by phosphorylating hexokinases HK1 and HK2, the glycolysis rate-limiting enzymes.^89^ High glycolysis results in elevated production of lactate and protons, leading to the acidification of the local primary tumor microenvironment that can result in further activation of transforming growth factor-β (TGF-β)^90^ to repeat the EMT-MET cycle (Fig. 1).

How metastatic foci learn to thrive in a new environment? One possibility is that, before migration, the primary tumor cells precondition the systemic circulation and metastatic niche through exosomes and cytokines to manage immune cell encounters^91^ (Discussed methods of preconditioning in detail below) (Fig. 1). Despite preconditioning, the EMT pathway has numerous obstacles in every step of metastasis such as vascular entry and exit. Some of these hurdles in the EMT pathway are being explained by an emerging alternate BMW transport pathway. The remainder of the review will be focusing mainly on the BMW pathway of metastasis while comparing the EMT pathway at critical discussion points below.

## Blebbishield metastatic-witch (BMW) pathway for metastasis

The reversible EMT pathway to metastasis is well-worked out in the context of TGF-β signaling as described above. However, the signaling intermediates such as the TAK1 exon-12 variant (not wild-type),^38^ and epigenetic remodeling by altered PRC2 complex function^35,92^ compromise the cell death-inducing ability of TGF-β signaling. However, recent studies on metastasis in the context of transcriptome clearly indicates the existence of a cell death process-dependent pathway for metastasis.^24^ Multiple forms of cell death processes have roles in metastasis, such as reversible apoptosis through blebbishield emergency program,^18,93^ necroptosis,^84,85,94^ ferroptosis,^95^ pyroptosis,^95^ and NETosis (DNA extruded neutrophil extracellular trap forming cell death type^96–99^). Many of these cell death processes have one property in common: reversibility of cell death.^16,18,93,100–102^

Among all the reversible cell death processes that have been documented, blebbishield emergency program stands out, as it has very characteristic apoptotic morphology by forming apoptotic blebbing^18^ and apoptotic body formation, followed by reconstruction of the blebbishields from apoptotic bodies.^18,93,100^ The reconstruction of apoptotic cells into blebbishields and subsequent spheroid state is driven by membrane fusion between blebbishields-to-blebbishields or blebbishields-to-mitotic cells^18,100^ or blebbishields-to-immune cells to gather multiple copies of the genome under one membrane.^93^ This fusion behavior generates a poorly or less differentiated/polarized transformed state marked by spheroid formation.^18,21,93,100,103–106^ The spheroid state later undergoes differentiation/polarization and exhibits a well-marked exit phase^93^ that generates and releases polarized individual cancer cells outside the spheroid with properties such as migration,^93^ genomic instability,^93^ protected mitochondria (less mitochondrial outer membrane permeabilization/MOMP) with enhanced glycolysis,^19^ immune evasions, and increased metastasis^93^ (Fig. 2). In addition, the genetic signature of a transformed state of blebbishield emergency program^93^ shares signatures with EMT pathway, ferroptosis reversal, necroptosis inhibition and anastasis (Discussed below). Therefore, blebbishield emergency program can be considered as a prototype for cell death-driven metastasis by the BMW pathway.

## Malignant transformation of the witch that prepares to move

Cellular transformation (acquiring spheroid structure with EMT-like poorly or less differentiated/polarized state) and accumulation of genomic instability (numeric and structural) are required for tumorigenesis and metastasis of cancer cells, and K-Ras/MAPK signaling emerges as a central driver of these pivotal events^19,93,104^ (where the contribution of other oncogenes such as Src, Myc, H-Ras, N-Ras, and others also play a role depending on cellular/tissue type context-dependent manner). When a cell undergoes cellular transformation for the first time, the level of genomic instability will be lower and can be classified as neoplastic transformation.^21^ Metastasis is fueled by genomic instability^4^ and malignant transformation where pre-transformed cancer cells (that transiently lost the EMT-like poorly differentiated state) undergo further rounds of cellular transformation to accumulate severe genomic instability.^21^ Endoreplication,^107^ virus^108^ or BUB1 and or MAD2 mutations (Fig. 2), cell death process^93^-driven cell fusion during transformation can augment genomic instability.

Studying malignant transformation (soft-agar clonogenic assays) is a long process. This hampered the discovery of short-term events that lead to cellular transformation. A recent discovery of blebbishield emergency program uncovered the requirement of an apoptotic process for cellular transformation by reversal of morphological and biochemical apoptosis^7,18,19,21,93,100,103–106,109–113^ (Fig. 2). Upon initiation of apoptosis, glycolysis plays a pivotal role to avert secondary necrosis during the reversal of apoptosis^19^ (Fig. 2). This is a very essential step, as necrosis or secondary necrosis compromises the reversibility of the cell death process. The glycolytic metabolic requirement can be well coordinated by c-Src, which phosphorylates hexokinase-1 (HK1) at Y732 to promote glycolysis, tumorigenesis,^114^ and metastasis.^89^ K-Ras oncogenic product also promotes glycolysis through oligomerization during blebbishield formation^19^ (Fig. 2). In parallel to aversion of secondary necrosis, the cells have to neutralize the intrinsic and extrinsic apoptotic signals to comfortably reverse apoptosis. For this purpose, the apoptotic cells utilize the K-Ras oncogene. Reactive oxygen species (ROS) in apoptotic cells drive sustained activation of K-Ras and PKCs.^104,113^ The extrinsic apoptotic signals are compromised by c-IAP-1 and 2, intrinsic apoptotic signals are compromised by mitochondrial outer membrane permeabilization level and XIAP combats caspase cascade^19^ (Fig. 2). K-Ras drives the reversal of apoptosis through the internal ribosome entry site (IRES)-mode of translation of cellular inhibitors of apoptosis proteins (c-IAPs and XIAP)^19^ (Fig. 2). Interestingly, adenosine deaminase acting on RNA-1 (p110, ADAR1) protects anti-apoptotic mRNAs from decay at cytoplasm^115,116^ and could explain how the game-changing anti-apoptotic factors such as XIAP, c-IAPs are readily converted to protein by IRES translation during apoptosis^19^ (Fig. 2).

The next important step in the reversal of apoptosis is disabling the p53-dependent transcriptional program, as p53 drives an array of proapoptotic genes, including GADD45A/B and Fas.^117^ Blebbishield emergency program is driven by the loss of p53 function (a major tumor suppressor known to date)^93^ and activation of K-Ras^104^ to result in increased metastasis.^93^ Consistently, metastatic tumors from multiple cancer types revealed that *TP53* and *KRAS* are more frequently mutated genes.^118^ It is worth stating that most mutations in *TP53* are of inactivating p53 function^118^ (a few are of gain-of-function type), and those in *KRAS*^118^ are of constitutively activating K-Ras^104^ to implicate the importance of blebbishield-mediated transformation in cancer metastasis.

Furthermore, blebbishield emergency program contributes to ploidy-level numeric chromosomal instability.^93^ Thus, rounds and rounds of malignant transformation (at least two successive rounds were demonstrated: blebbishield serial ejection (BSE-2)^18,93^) can lead to the selection of CSCs that are efficient in glycolysis^19^ with accumulated genomic instability.^93^ The ploidy-level genomic instability in blebbishield emergency program is primarily achieved through blebbishield-to-blebbishield^18^ or mitotic^100^ or immune^93^ cell fusion. While p53 defective cells usually harbor a high degree of genomic instability, p53 positive cells utilize timely suppression of p53 at the protein level during the transformed spheroid state.^93^ Furthermore, apoptotic DNA fragmentation and non-homologous end-joining (NHEJ) mode of DNA repair by K-Ras-PARP1/XRCC1/DNA ligase-3a axis^104,119^ can generate further structural genomic instability. K-Ras drives NRF-2-driven ROS neutralization to block further DNA damage and activate homologous recombination-directed DNA repair.^104^ Thus, K-Ras plays an inevitable role in blebbishield-mediated reversal of apoptosis and glycolysis, and the glycolytic by-product lactate, in turn, makes the apoptotic microenvironment acidic and promotes blebbishield formation and subsequent transformation of apoptotic cells^18^ (Fig. 2). While in this section we provided details on the key framework of blebbishield-mediated cellular transformation, the other essential aspects of blebbishield emergency program are described below in appropriate sections.

## Blebbishields vs. other forms of reversible cell death: preparing the Trojan horse

### Apoptosis

Why cancer stem cells need the process of apoptosis but not full-blown apoptosis for cellular transformation? It’s a very important question in the context of stemness and metastasis. Apoptosis initiates the loss of attachment with neighboring cells by pyknosis^18^ irrespective of their epithelial or mesenchymal state. Pyknosis is associated with AJ disassembly,^120^ loss of interactions between various integrins, E-cadherin, cleavage of β-catenin by caspase to a 72-kDa fragment^100^ which detaches active β-catenin from E-cadherin on plasma membrane interior to undergo nuclear translocation and initiate transcription. This helps to ignite VEGF transcription, a driver of blebbishield emergency program^100^ and vascular permeability.^121^ This explains why apoptosis is specifically required for metastasis because mitotic cells can also lose attachment with neighboring cells but have a lesser degree of caspase activation. The apoptotic cells have more advantage than mitotic cells towards metastasis because apoptosis unleashes the IRES emergency translation during the reversal of apoptosis (Fig. 2)^19,24^, which can be utilized by the apoptotic cells to translate VEGF (to drive blebbishield emergency program), c-Myc/N-Myc (to drive glycolysis and metastasis), and c-IAPs such as c-IAP-1/2, XIAP (to block death signaling and caspase-3).^19,100^ The apoptotic event prepares the cancer cells for drug resistance, stemness, and metastasis. Apoptotic cell’s fusion behavior^18,100^ is required for cellular transformation and is implicated in metastasis.^93,122^ Despite blebbishield emergency program conserves the mitochondria from depolarization,^19^ cells with defective p53 (TP53-FXR2 fusion) and depolarized mitochondria were shown to undergo transformation.^117^ This suggests that mitochondria depolarization is not a limiting factor of cellular transformation. Apoptosis is known to activate TGF-β through LAP-processing of the ligand.^123^ In addition, activation of MMP-2 and 9 during apoptosis^124^ and cleavages of vitronectin and fibronectin are pre-requisites for metastasis.^125^ Both activation of TGF-β and MMPs can be attributed to the extensive remodeling of the extracellular matrix during apoptosis.

### Necroptosis

Necroptosis is a hybrid form of cell death that has overlapping signatures of both apoptosis and necrosis. Necroptosis can kick in when the caspase-8 function is impaired during apoptosis^126^ and explains why these two cell death modalities overlap to a significant extent. Similar to blebbishield-mediated reversal of apoptotic cancer cells, necroptosis is also recently found to defy cell death by using ESCRT-III components such as charged multivesicular body protein 4B (CHMP4B) and CHMP2A.^127^ Necroptosis of endothelial cells by amyloid precursor protein (APP) from tumor cells facilitate extravasation of circulating tumor cells (CTCs) to promote metastasis.^85^ Furthermore, TGF-β signaling component TAK1 deficiency is required for endothelial cell necroptosis.^84^ However, necroptosis is not demonstrated to contribute to spheroid formation, but the spheroid cell may die through necroptosis in response to therapeutics. Therefore, necroptosis could be more related to the EMT pathway of metastasis at the extravasation stage. Importantly, endothelial cell necroptosis subverts the diapedesis mode of extravasation.

### Ferroptosis

Ferroptosis is a non-apoptotic, iron-dependent form of cell death where iron, ROS, and lipid peroxidation play a major role.^16^ Intracellular iron accumulation is primarily done through uptake of Fe^3+^ through transferrin-transferrin receptor (TFR1) endocytosis.^128^ This intracellular iron pool is stored in ferritin heavy and light chains.^128^ Furthermore, the cystine transport-GSH-GPX4 axis inhibits lipid peroxidation and is linked to ferroptosis regulation.^128–130^ Therefore, iron chelating agents, lipid peroxidation inhibitors, and anti-oxidants were able to reverse ferroptosis.^16^ This form of cell death gains more importance in iron-loaded tissues such as liver or lymph nodes. Increased ferroptosis is known to release prostaglandin-E_2_ (PGE_2_), an immunosuppressive agent^130^ therefore, a high ferroptosis environment may trigger immune cell anergy through PGE_2_.

### NETosis

Neutrophil extracellular trap (NET) formation is a morphologically unique form of cell death where the hyper-citrullinated decondensed chromatin is extruded outside the cell to form net-like structures.^131^ NET is initially shown in neutrophils in the context of interferon and pathogen encounter^132,133^ and later in the context of Raf/MEK/ERK pathway^134^ and in other cell types.^135^ Intriguingly, this form of cell death is silenced by IL-6.^136^ NETosis is specifically linked to metastasis at the extravasation stage of circulating tumor cells.^97^

### Anastasis

Anastasis, a reversible form of apoptosis, has an enrichment of genes related to the EMT pathway and is driven by TGF-β and Snail.^17^ This raises the possibility that the EMT pathway could use a cell death-driven mechanism for metastasis but yet to be worked out. Furthermore, anastasis generates individual survived cells rather than a spheroid after the commencement of apoptosis.^17^ Much has to be worked out in the context of anastasis involvement in metastasis to relate this reversible cell death to EMT and or BMW pathway of metastasis.

### What drives reversible cell death?

While the reversal of ferroptosis, apoptosis, and necroptosis is well-documented, it is not yet known whether necroptosis, ferroptosis, and blebbishield formation (from apoptotic cells) utilize the same overlapping mechanism to defy cell death or not, but these cell death reversals were linked to tumorigenesis^18,137,138^ and metastasis.^85,93,139^ Blebbishield formation is linked to dynamin-dependent endocytosis^100^ and inhibiting dynamin-dependent endocytosis during blebbishield formation enhances death through proper complete apoptosis.^100^ Similarly, ESCRT-III is linked to endosomes [to form multivesicular bodies (MVB)],^140^ and necroptotic bubble formation (analogous but different structures to the apoptotic blebbing that exposes phosphatidyl serine).^141^ Inhibiting ESCRT-III component CHMP4B enhances full-blown necroptosis.^140,141^ Strikingly, ferroptosis is also dependent on endocytosis where transferrin receptor TFR1 takes iron inside the cells with transferrin^142,143^ and suppression of transferrin receptor by HUWE1 E3 ubiquitin ligase inhibits ferroptosis.^144^ Finally, the molecular signature of anastasis also reveals the alterations in endocytosis.^17^ Therefore, an endocytosis process potentially drives reversibility of these cell death processes albeit using different types.

Interestingly, inhibition of hyaluronan endocytosis by using monoclonal antibodies inhibited prostate cancer cell metastasis, in particular to the lymph nodes.^145^ This study suggests that the reversible cell death-driven BMW pathway prefers the lymphatic route of metastasis (see below). Notably, the metastasis suppressors such as NME, KAI, MTSS1, and KISS1 also have been shown to operate on endocytosis regulatory mechanisms.^146^ For example, NME1 and NME2 regulate dynamin-dependent endocytosis in the context of metastasis.^147^ These facts indicate the pivotal role of endocytosis in the context of the BMW pathway of metastasis.

## DAMPs: find-me and eat-me signals as the devil’s barbeque fragrance bait

The therapy-induced or natural cell death milieu within the tumor microenvironment is not usually uniform as it includes apoptotic cells with secondary necrosis, necroptotic cells, necrotic cells, and live non-apoptotic cells. The necrotic (as well as secondary necrotic) cells release an array of damage-associated molecular patterns (DAMPs), which help to attract immune cells to the local environment (find-me signals) and clear the apoptotic site by phagocytosis of apoptotic bodies (through eat-me signals such as phosphatidyl serine) (Fig. 2). The DAMP signals include but not limited to HMGB1, S100 proteins, hyaluronan, heat-shock proteins (HSPs), ATP, calreticulin, uric acid, and IL-1α.^148^ Of these DAMPs, IL-1α is known to promote malignant transformation,^149^ angiogenesis, and metastasis via its interaction with platelets.^150^ Furthermore, S100 family proteins released by necrotic cells are also linked to cell migration and metastasis.^151,152^ Hence DAMP-mediated attraction of immune cells such as cytotoxic T-cells, phagocytes, and APCs are clearly not helping to clear the cell death scene, rather it makes the scenario worse and prometastatic. Cytosolic genomic DNA was found to trigger immune evasion and inflammation-dependent cancer in STING deficient mice with Ras oncogene.^153^ However, recurrent chromosome segregation errors promote metastasis through STING-pathway^154^ (Fig. 2). Such chromosomally unstable cells disable the immune system and promote metastasis through extracellular hydrolysis of cGAMP through the ectonucleotidase ENPP1.^155^ Macrophages that were exposed to phosphatidyl serine (from apoptotic cells) secrete TNF-α, CCL4, CCL5, and IL-6 to drive dead/dying cell-induced tumor growth enhancement by inflammation, and neutralizing antibodies against these factors abrogated the dying cell-induced tumor growth enhancement.^156–158^ Therefore, DAMPs not only promote metastasis but also promote primary tumor growth in part through the recruitment of immune cells to the local microenvironment when active cell death is in progress.

## Serpentine filopodia of the blebbishields vs. immune cell cops: let the blebbishield arms do the talking

Recruitment of immune cells to the tumor microenvironment with active cell death is an ideal condition for phagocytic clearance of apoptotic bodies by phagocytes. However, the dying tumor cells must overcome eat-me signal-activated phagocytes to undergo metastasis or to survive. This can be achieved by the “fuse-to-me” signals exposed to dying tumor cells. In blebbishield emergency program, the fuse-to-me signals identified were cholesterol and endocytosis-driven serpentine filopodia (long and stout villi-like cellular projections with a bulb that tie the apoptotic bodies together to drive membrane/cell fusion)^18,100^ implicating active cdc42 in the fuse-to-me process (Fig. 2). Adhesion-zippers (different from adherens junctions) are anti-parallel filopodia structures from adjacent cells with β-catenin-α-catenin linkers between surface E-cadherin and the internal actin cytoskeleton facilitates cell fusion.^100,159^ Therefore, activation of cdc42 plays a central role in the fuse-to-me event. XIAP downregulation during apoptosis of CSCs^19^ can elevate cdc42 expression to regulate filopodia formation.^160^ PTEN-ε suppresses metastasis by regulation of filopodia formation^161^ to highlight the role of filopodia in the metastatic process. Re-expression of XIAP through IRES translation can reduce cdc42 activity/expression to serve as a negative feedback loop because XIAP degrades cdc42 through the ubiquitin-proteasome system.^160^ In this context, serpentine filopodia is known to get shortened to microvilli once apoptotic cells completed major membrane fusion events and blebbishields are formed.^100^ Interestingly, fusion-capable blebbishields exhibit selective suppression of p53 with a consequent promotion of Galectin-3.^93^ Galectin-3 is capable of inducing an apoptotic state in immune cells and at the same time capable of promoting K-Ras signaling within cancer cells through β3 integrin binding.^162^ Such human apoptotic cells capable of suppressing p53 are demonstrated to fuse with human immune cells, express increased K-Ras activation, generate increased metastasis in the mouse model, and form hepatosplenomegaly with low p53.^93^ Thus, the fuse-to-me signals on blebbishields override the phagocytosis checkpoints to achieve metastasis. In this context, expressing mouse CD47 (another fuse-to-me signal^163^) in human cancer cells elevated the metastatic potential in the mouse model and notably increased the lymph node size.^164^ This suggests the lymphatic route of metastasis when CSCs fuse to immune cells (Fig. 2). If p53 expression is not compromised, p53 mutations were found to get enriched in metastatic cells, especially after cytotoxic therapy.^165,166^ Therefore, disabling wild-type p53 transcriptional program could be the key to facilitate dying cell to immune cell fusion through various fuse-to-me signals.

## Migration: the blebbishield metastatic-witch (BMW) transport vs. classical EMT crawling

In general, reversible/hybrid-EMT is considered the major mechanism for metastatic migration.^1^ However, VEGF/VEGFR-driven blebbishield emergency program^18,100^ offers a simple solution to metastasis by blebbishield-immune cell fusion, where the migratory capacity and the homing signals of immune cells can be used by blebbishield-immune cell hybrids to reach target organs^93^ (Fig. 2). For example, blebbishield-immune cell fusion results in the increase in IGFBP5 and intense migration compared to the parental cancer cells in vitro and localization to liver and spleen in vivo^93^ in bladder cancer.^167^ Furthermore, cancer cell-macrophage fusion promotes metastasis^168^ or, inhibition of VEGFR-1 using neutralizing antibody impedes treatment-induced metastasis.^169^ Notably, neutralizing antibodies to VEGF/VEGFR2 block blebbishield emergency program.^100^ Furthermore, the BMW model offers an ideal explanation that the fusion with immune cells or apoptotic CSCs generates a wide range of chromosomal instability^93^ that can potentially result in polyclonality/heterogeneity at the metastatic site upon exhibiting the exit phase of blebbishield emergency program where multiple individual cells emerge from a single spheroid with genome variations.

In the context of metastasis, there exists two models of migration, such as the sequential progression model where the cancer cells invade the basement membrane (Fig. 1) and/or local lymph nodes (Fig. 2) before reaching the actual target organ for metastasis, or a clone within primary tumor directly enters the local angiogenic bloodstream to reach the target organ.^170^ The cells that take the sequential progression model of metastasis through the lymphatic route or directly move to secondary lymphoid organs are probable cancer cell-immune cell hybrids that reversed the cell death process^93,171^ utilizing the BMW transport route of metastasis. For example, macrophages have homing signals for lungs and other tissues and hybrids of tumor cells-macrophage fusion preferentially metastasize to lungs^171^ or fusion of tumor cells with bone marrow-derived cells during bone metastasis.^172^ One of the main reasons for the lymphatic route preference of the BMW pathway is the fact that lymph provides a cell death resistance mechanism through high GSH, GPX4, and oleic acid-directed ferroptosis resistance, with reduced oxidative stress and lesser free iron compared to the hematogenous route of metastasis.^139^ In BMW transport, the apoptotic CSCs can be considered as the passenger and the immune cells that fuse to apoptotic cancer cells can be considered as the transport vehicles (broomstick of the witch). Inhibition of macrophage M2 polarization by all-trans retinoic acid (ATRA) prevents lung metastasis and suggests that M2 macrophages are the BMW transport vehicles in osteosarcoma metastasis.^173^ Blebbishield emergency program results in transient loss of E-cadherin to β-catenin interaction^24,100^ suggesting the existence of an EMT-like program in BMW transport. Although EMT is tightly linked to E-cadherin loss, the lymphatic route taken by the metastatic cells strongly suggests that these cells utilize BMW transport for metastasis. To add more support to the BMW transport, multiple chemotherapeutics cause a sharp increase in the intravasation of tumor cells following chemotherapy.^174^ Notably, tumor-derived VEGF-C promotes lymphangiogenesis towards cancer cells to promote the lymphatic route of metastasis,^175–177^ and promotes immune tolerance by disabling/eliminating CD8^+^ T-cells.^178^ In colon cancer and melanoma, the cancer-macrophage fusion hybrids exhibited increased motility and invasion before metastasis.^171,172,179,180^ In breast cancer, the apoptotic cells fuse spontaneously with multipotent stromal cells to acquire increased migration and achieve metastasis.^122^ Androgen receptor could favor the EMT crawling mode of metastasis over BMW transport as it augments hematogenous metastasis and reduces lymphatic metastasis in renal cell carcinomas.^181^ In all, the BMW transport mechanism potentially offers a quick, efficient, and immune cell fusion-dependent method for metastasis compared to the classical EMT-driven crawling model of metastasis.

## Preconditioning the metastatic niche vs. preconditioning the passenger

**Fig. 4:**
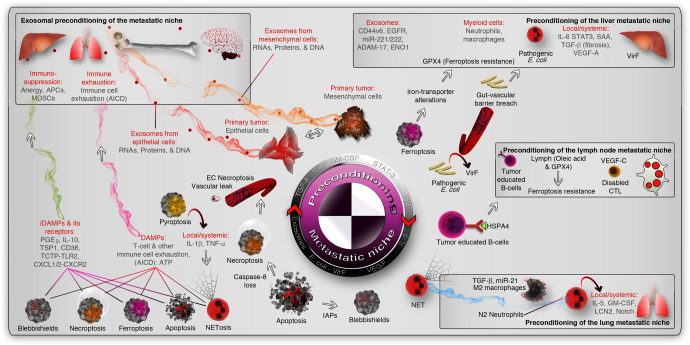
Preconditioning of the metastatic niche. The epithelial and mesenchymal cells precondition the metastatic niche (marked by inset boxes) by cytokine or chemokine secretion or primarily through exosomes (red and orange smoky trails, respectively. The liver, lymph node, and lung metastatic niche were shown as examples (inset boxes). Microbial preconditioning of the liver niche is shown as an example (pathogenic vir-F containing *E. coli*), and exosomal and immunological preconditioning is also shown in the liver niche. In the lymph node niche, tumor-educated immune cells (B-cells exposed to HSPA4 from tumors) or VEGF-C induced exhaust of cytotoxic T- cells (CTLs) or lymph-protected tumor cells serve as preconditioning mediators (non-exhaustive list of mechanisms). In the lungs, polarized immune cells (into pro-tumor) precondition the niche by cytokines and lipocalin-2 (LCN2). In the cell death context, DAMPs or iDAMPs induce cytotoxic immune cell exhaustion by AICD or immunosuppression, respectively (magenta and green smoke trails, respectively). AICD activation-induced cell death, EC necroptosis endothelial cell necroptosis, MDSCs myeloid-derived suppressor cells, APCs antigen-presenting cells, PGE_2_ prostaglandin-E_2_, TSP1 thrombospondin-1, TLR2 Toll-like receptor-2, IAPs inhibitor of apoptotic proteins, NET neutrophil extracellular trap

Frequently, the locations where the cancer cells are attracted to colonize as metastatic foci are depots of killer immune cells. Examples include regional draining lymph nodes, liver, and spleen, which are storehouses of cell-mediated and or humoral immune cells. The cancer cells must adapt to evade the cytotoxic and humoral immune cells to establish metastasis in such organs or remotely precondition the metastatic site so as to disable the immune attack (or enable immune checkpoints). In the case of reversible EMT-mediated metastasis, prior to metastasis to the liver, the liver accumulates myeloid cells, undergoes fibrosis, and the hepatocytes reprogram the niche by activating IL-6/STAT-3 to produce SAA^182^ (Fig. 4). Alternatively, tumor cells release exosomes that educate the immune system (systemic or local; depending on the surface molecule expression such as ANXA6 on exosomes or the proteome and DNA/RNAs/miRNAs that are stored within exosomes)^183^ (Figs. 1, 4). Furthermore, bone marrow-derived cells precondition the metastatic site before the metastatic cancer cells arrive.^4^ This is achieved by the polarization of immune cells towards tumor-associated immune phenotypes (M2 macrophages, N2 neutrophils, MDSCs, and so on^7^). Very often, this polarization is associated with defective antigen presentation by tumor-associated macrophages (TAMs), and enabling its antigen presentation with Toll-like receptor agonists reignited the anti-tumor immunity.^184^ Furthermore, in a lung cancer model, miR-21 suppresses the anti-tumor action of tumor-associated macrophages.^185^ In the context of tumor-associate macrophages in the lungs, M2 macrophages precondition the lung niche for metastasis (Fig. 4). TGF-β a M2 macrophage polarization promoting factor^7^ protects mammary adenocarcinoma cells at the lung metastasis site.^186^ Smad4 degradation blocks TGF-β signaling to inhibit lung metastasis,^187^ to envisage the importance of TGF-β-mediated preconditioning for lung metastasis. Neutrophils on the other hand, facilitate lung metastasis by preconditioning the lungs through IL-5, GM-CSF,^188^ lipocalin-2 (*LCN2*),^189^ and notch signaling^190^ (Fig. 4). Often, lymph node metastasis serves as the tumor-specific immune tolerance mechanism for subsequent distant metastasis.^191^ Tumor-educated B-cells produce pathogenic IgGs to bind HSPA4 to precondition lymph node niche and promote lymph node metastasis^192^ (Fig. 4). VEGF-C in the lymphatics disable/eliminates the cytotoxic T-cells and protects melanoma cells from immune system^178^ (Fig. 4). Importantly, the lymph (oleic acid, low iron, GSH-GPX4)-dependent ferroptosis resistance mechanism discussed above^139^ could favor VEGF-C-dependent lymph node metastasis.

It should be noted that many xenograft studies use human cells implanted in mice to study metastasis where tumor cell induced preconditioning might not be accurate. This is because the human genome has a huge miRNA cluster on chromosome-19 (C19MC) and which is not present in the mouse genome.^193^ C19MC is potentially regulated by adipogenic enhancer regulators like CEBPB^194,195^ or by alterations in K-Ras and p53 signaling.^196^ C19MC miRNAs are implicated in cancers and can be transferred to distant sites through exosomes.^197,198^ Multiple exosome-delivered cargoes such as CD44v6/C1QBP complex (to promote fibrosis),^199^ EGFR (to activate HGF),^200^ miR-221/222 (from colorectal cancer),^201^ ADAM-17(to activate E-cadherin cleavage),^202^ ENO1 (to promote α6β4 integrin), precondition the liver metastatic niche to promote metastasis from various type of primary tumors (Fig. 4). Conversely, murine colorectal cancer cell-derived exosomes in fact inhibit peritoneal metastasis through NK cells and IFN-γ in mice^203^ suggesting that, not just exosomes but the exosomal content matters in preconditioning metastatic niche. An example in the context of preconditioning of brain metastasis niche is the exosomal protein ‘cell migration-inducing and hyaluronan-binding protein’ (CEMIP) which preconditions the CD31^+^ brain vasculature through remodeling diapedesis and neuroinflammatory genes.^204^

Interestingly, tumor-resident *E. coli* with virulent factor VirF can do the preconditioning work in the liver when it breaches the gut vascular barrier^205^ (Fig. 4). In addition, Mycobacterium tuberculosis can suppress Th1 immunity and promote lung metastasis.^206^ These facts open up the possibility that other microbes could determine preconditioning of metastatic niche. This possibility is further reinforced by the finding that multiple human tumor types such as breast, bone, pancreas, glioblastoma multiforme, ovary, lung, and melanoma harbor 16 S RNA signatures of an array of intracellular Gram-negative and Gram-positive bacteria matching metabolic and tumor sub-types.^207^ In addition to preconditioning, microbes also contribute drug resistance. For example, *Mycoplasma hyorhinis* can metabolize and inactivate the chemotherapeutic agent gemcitabine by its cytidine deaminase (CDD_L_) enzyme.^208^ On the other hand, *Fusobacterium nucleatum* utilizes the TLR4-autophagy axis to suppress apoptosis.^209^

In BMW transport, apoptotic CSCs fuse with immune cells to generate hybrid cells so as to acquire protection from immune surveillance and phagocytosis because the immune cell component of the hybrid already exhibits immune tolerance (by displaying self-antigens), and the cancer cell component of the hybrid offers escape mechanism from activation-induced cell death (AICD) and clonal deletion using blebbishield emergency program.^93^ This phenomenon can either induce metastasis by transporting cancer cells to a metastatic niche like liver and/or spleen^93^ or can induce myeloid or lymphoid leukemias,^210^ depending on the immune cell fusion partner. Dying tumor cells induce T-cell exhaustion^211^, which can further contribute to systemic energy towards cancer cells. The immune cell exhaustion can potentially be induced by the dying cell components (DAMPs: such as ATP, HMGB1) acting as immunogenic determinants^212–216^ to promote activation-induced cell death (AICD) of immune cells (Fig. 4).

The next major mechanism of preconditioning the metastatic niche in the context of the BMW pathway (by different cell death modalities) is by inducing immune suppression. This is largely achieved by the release or activation of inhibitory damage-associated molecular patterns (iDAMPs) such as PGE_2_,^215^ IL-10,^217^ thrombospondin-1 (TSP1/THBS1),^217^ CD36,^217^ TCTP-TLR2,^218^ CXCL1/2-CXCR2^218^ (Fig. 4). These iDAMPs promote immunosuppression and induction of pro-tumor immune cell polarization that can support preconditioning of metastatic niche. Interestingly, iDAMPs dominate DAMPs to suppress immunogenic cell death. For example, when DAMPs such as calreticulin, HSP70, and HMGB1 were in action from dying cells, the iDAMP PGE_2_ suppresses the immunogenicity of the cell death.^215^

Preconditioning by cell fusion in the BMW pathway enables stealth dissemination of pancreatic ductal adenocarcinoma cells to the liver, spleen, and lungs after fusion with patient macrophages.^168^ Interestingly, the macrophage-tumor cell fusions exhibited ploidy-level genomic instability^219^ similar to blebbishield-immune cell fusion,^93^ and contributed to poor prognosis.^168^ Therefore, in BMW transport, the passenger (cancer cells) is preconditioned by fusion with immune cells to overcome the hurdles posed by the metastatic niche and by the lymphatic/metastatic route.

The preconditioning not only combats the immune system at the metastatic niche, but also suppresses anti-tumor immunity systemically in most cases, at least during the metastatic duration. This stage is presumably enriched with circulating tumor cells in blood or in lymphatics, as mesenchymal cells in the case of EMT pathway or as a cancer cell-immune cell hybrid in the case of BMW pathway. Additional reversible cell death modes may add more complex phenotypes in the circulation.

## Establishing metastasis at distant sites: let my claws cling on

While reversible/hybrid-EMT and BMW transport pathways are able to take the cancer cells to the premetastatic niche, settlement of tumor cells at metastatic sites requires additional factors for colonization. In the case of reversible/hybrid-EMT, MET conversion mediates the settlement. This is demonstrated by the fact that, migrating cancer cells (in ascites) that exhibit epithelial features are able to rapidly establish tumors at sites other than the primary tumor.^220,221^ However, there are metastatic cancers with a mesenchymal phenotype.^55^ This is in part mediated by alternative cell adhesion factors. Cell adhesion molecules like vitronectin, CD44-hyaluronan, fibronectins, complement component-C3, etc., mediate target site selection for metastatic colonization^3,125^ (Fig. 1). Furthermore, activation of αvβ3 integrins promotes the migration of premetastatic cells toward vitronectin and fibronectin for metastatic colonization in lungs.^222^ Interestingly, plakoglobin-directed CTC aggregation offers resistance to apoptosis and rapid establishment of metastatic colonies^223^, suggesting the importance of cell death resistance over EMT in establishing metastatic colonies. The most notable aspect of target site selection is often determined by the point of vascular exit. Lung being the most vascularized tissue, the exit of CTCs from alveolar vasculature is a key step that is driven by neutrophil NET formation by CTC secreted cathepsin-C through IL-1β.^98^ Sox-2 downregulation or promotion^45^ could be a key to initiate MET, which is known to promote MET and predict liver and lymph node metastases.^224^ Surface E-cadherin, in addition to VEGFR2 and PD-L1, marks cells capable of establishing tumors away from the primary tumor in the case of bladder cancer.^220^ Thus, MET would be required in the case of EMT-mediated metastatic migration to expose E-cadherin on the surface. In cells that undergo MET during metastatic colonization, a transcriptional program of c-Myc is pivotal as it promotes E-cadherin expression.^24^ Often, a mere expression of E-cadherin in the cell may not be sufficient because the ascites-derived bladder cancer cells have distinct population of cells with internal E-cadherin expression but has impaired surface E-cadherin expression behave inferior in tumor aggressiveness.^220^

In the case of BMW transport, the metastatic colonization might be achieved through polarization of cancer cells through the exit phase of blebbishield emergency program^93,100^ where calcium signaling and VEGF mediates polarization possibly through re-expression of c-Myc.^18^ In the case of BMW transport, the metastatic target selection molecules are mostly homing signals of the immune cell fusion partner. In macrophage-mediated BMW transport, the hybrid cells express a set of acquired adhesive molecules such as vitronectin and collagen XXIII, which are offered by macrophages.^171,225^

Platelet-derived autotaxin interaction with αvβ3 integrins on cancer cells directs bone metastasis.^226^ More interestingly, osteogenic cells supply calcium to cancer cells through gap junctions and promote bone micrometastasis.^227^ In brain metastasis of breast cancer cells, a combination of high cholesterol and low triacylglycerols (TAGs) were found. In this context, fatty acid synthase is required for breast cancer metastasis to the brain.^228^ Lipid rafts drive metastasis in the contexts of EMT^229^ and blebbishield emergency program.^18,93,100^ Cancer cells upregulate Amigo2, which facilitates liver endothelial cell attachment and liver metastasis.^230^ Filopodia formation results in increased lung colonization of cancer cells.^160^ ATF-4 and Nrf-2 dependent HO-1 expression is required for lung metastasis as ATF-4 deficiency impaired lung metastasis.^231^ Notably, blebbishield emergency program promotes HO-1 at the protein level in metastatic colonization sites (liver and spleen) and at the transcription level during a transformed state.^93^ HO-1 in tumor-associated macrophages promotes metastasis by extreme heme catabolism, immunosuppression, angiogenesis, EMT, and prometastatic niche conditioning.^232^ Therefore, different signals govern different target site selection for metastatic colonization irrespective of the EMT or BMW pathways.

## EMT and BMW pathway metastatic engines: identical or different?

**Fig. 5:**
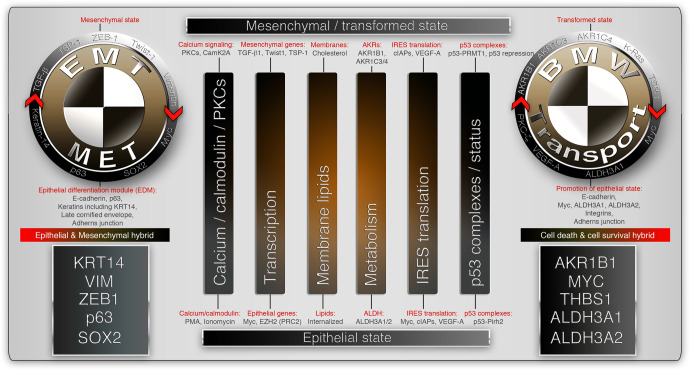
Similarities between EMT and BMW pathways of metastasis. The non-exhaustive common signatures associated with metastasis in the context of EMT and BMW pathways are represented. The mesenchymal state of the EMT pathway is analogous to the transformed state of the BMW pathway and are executed by similar biological functions but with different tools (Top side of the figure). Likewise, the reversible epithelial state of the EMT pathway is analogous to the exit phase of blebbishield emergency program (BMW pathway) and are executed by similar biological functions but with different tools (Bottom side of the figure). The main biological functions shared by EMT and BMW pathways are represented by vertical bars. The key molecules involved in reversible/hybrid-EMT is shown in the black box (far left) and the ones involved in reversible or cell survival and death hybrid is shown in the black box (far right). PKCs protein kinase-C, TSP1 thrombospondin-1, c-IAPs cellular inhibitors of apoptotic proteins, AKRs aldo-ketoreductases, VEGF-A vascular endothelial growth factor-A, PRMT1 protein arginine methyltransferase-1, ALDH aldehyde dehydrogenases, PRC2 polycomb-repressive complex

An important question in the context of metastasis is, how similar the reversible/hybrid-EMT and BMW pathways are? As both the pathways do one common task, metastasis, it is conceivable that these pathways must have some overlapping parts in their metastatic engine. We list out six major overlaps which is non-exhaustive (could have more): (1) Calcium calmodulin signaling, (2) Transcription, (3) Membrane lipids, (4) Metabolism, (5) IRES translation, and (6) Status of p53 function (Fig. 5).

Calcium/calmodulin (CAMK2) signaling acts in reversible hybrid-EMT pathway^44,51^ and fits in the BMW pathway by regulating apoptosis through death-activated protein kinase (DAPK).^233^ While calcium/calmodulin signals through protein kinase-C (PKC), PKC-ζ is a common player in reversible EMT^234^ and BMW^93,100,113^ pathways (Fig. 5). While PKC-ζ and calcium/calmodulin signaling fit very well in epithelial state context, a different isoform of PKC-ζ (potentially a constitutively active form) is involved at least in BMW pathway.^93,100,113^ In the context of transcription, the transformed state of BMW pathway^93^ and the mesenchymal state^235,236^ of EMT pathway share transcription of TGF-β target, thrombospondin-1/TSP1/*THBS1* (Fig. 5). Furthermore, global transcription change is directed by alterations in PRC2 components and Twist1 in this context.^237–240^ Membrane lipids such as cholesterol show commonalities between EMT and BMW pathways where cholesterol is required for blebbishield membrane fusion^18^ and required to protect EGFR from degradation^241^ and cell fusion^242^ in the context of EMT. Notably, lipid-stained fusogenic membrane externalization and internalization mark the transformed state versus exit phase (recovered cells) respectively in blebbishield emergency program^100^ (Fig. 5). Importantly, genes such as aldo-ketoreductases (*AKR1B1, AKR1C3, AKR1C4*) with EMT-inducing ability^243–246^ were specifically transcribed during a transformed state of blebbishield emergency program^93^ (Fig. 5). On the other hand, EMT inhibiting or epithelial cell expressed metabolic genes such as *ALDH3A1*^247^ and *ALDH3A2*^248^ were also specifically expressed in a transformed state of cancer cells in BMW pathway.^93^ IRES translation is also shared by both EMT^249^ and BMW^19^ pathways (Fig. 5). Most importantly, p53 status and the complexing pattern is also shared by both EMT and BMW pathways, where a p53 complex with arginine methyltransferase PRMT1 regulates EMT^250^ and suppress apoptosis. In the BMW pathway, p53 is selectively suppressed.^93^ In the reversibility of EMT, p53 complexes with Pirh2 to degrade Twist1 to inhibit EMT^251^ (Fig. 5). These studies highlight the fact that both reversible EMT and BMW pathways utilize similar mechanisms to promote metastasis. Just like co-expression of KRT14, VIM marks the reversible hybrid-EMT with the expression of additional epithelial genes such as p63, Myc, and Zeb1, cells exhibiting reversible death also could have co-expressing signatures such as AKR1B1, ALDH3A1^93^ in addition to the GPX4 ferroptosis inhibitor. Therefore, both reversible EMT and BMW pathways indeed have similarities to do the common task of metastasis.

**Fig. 6:**
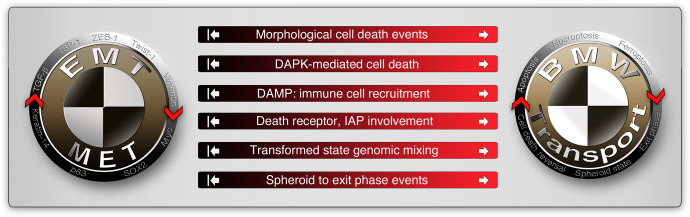
Differences between EMT and BMW pathways of metastasis. The non-exhaustive signatures that discriminate EMT and BMW pathways are illustrated. The white arrows on the red side of the horizontal bars indicate that these events are involved in the BMW pathway of metastasis. The white inhibited arrows on the black side of horizontal bars indicate, that these events are less or not at all involved in the EMT pathway of metastasis. Note that these events may happen in a non-metastatic context in both pathways. DAPK death-activated protein kinase, IAP inhibitor of apoptotic proteins (c-IAPs-1/2, XIAP), DAMP damage-associated molecular patterns

The next important question is how different the reversible/hybrid-EMT and BMW pathways are? A marked difference between the reversible/hybrid-EMT pathway and BMW pathway is the involvement of morphological and reversible cell death processes such as apoptosis, necroptosis, and ferroptosis and potentially other forms of cell death such as anastasis (Fig. 6). One notable example is that, TGF-β induces DAPK expression, but apoptosis is suppressed by TGF-β signaling itself at the MOMP/cytochrome-c release level^33^ (Fig. 6). Lack of considerable involvement of cell death-related events such as altered or disabled death receptors,^52,252^ IAPs^253^ in the EMT pathway suggests the DAMP-mediated immune cell recruitment to the tumor microenvironment (Fig. 6) is a discriminating factor. Of note, the hybrid-EMT pathway is well capable of altering immune cell recruitment through other mechanisms such as cytokines, chemokines, and prostaglandins. Finally, in the spheroid state, genome mixing from multiple blebbishields followed by exit phase to release cells with varying genomic instability are unique to the BMW pathway compared to EMT pathway, despite cells with EMT may undergo spheroid-like aggregation in soft-agar assays. On the other hand, the BMW pathway can use EMT mechanisms to drive metastasis. For example, the death receptor CD95/Fas is known to promote EMT and metastasis.^254,255^ Among the prominent death ligands (FasL, TNF-α, and TRAIL), FasL stands out to support blebbishield emergency program in combination with Smac mimetic.^19^ Thus, the reversible cell death process is a major discriminating factor that distinguishes the BMW pathway from the EMT pathway. However, the possibility of a cooperation between BMW and the EMT pathway to establish metastasis is not ruled out yet.

## EMT and BMW pathway vulnerabilities as therapeutic targets

**Fig. 7:**
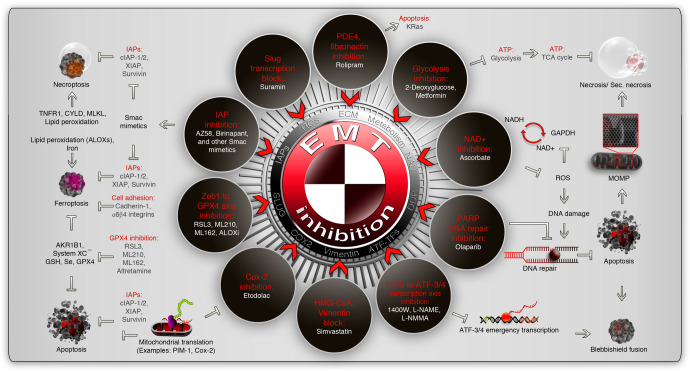
Therapeutic vulnerable points which are common to EMT and BMW pathways of metastasis. The common repurposed drugs (white fonts) that aim to inhibit EMT in the contexts of various biological processes (red font) are indicated within brown rings. The EMT inhibitory drug connection to various cell death pathways are indicated outside the brown rings. Note that the IAPs are involved in the reversibility of various cell death forms. Some pathways, such as NAD+ inhibition and PARP1 inhibition, are highly connected through DNA-damage response (DDR). See text for more details and caution statements. IAPs inhibitor of apoptotic proteins, TCA cycle tricarboxylic cycle, PDE4 phosphodiesterase-4, GPX4 glutathione peroxidase-4, iNOS inducible nitric oxide synthase, NAD nicotinamide adenine dinucleotide, CYLD cylindromatosis, GSH glutathione, MLKL mixed lineage kinase domain-like protein, PARP poly (ADP-ribose) polymerase, ATF activating transcription factor; ATP, adenosine triphosphate, GAPDH glyceraldehyde 3-phosphate dehydrogenase, ROS reactive oxygen species

The main goal of classifying the metastatic events into EMT and BMW pathways is to understand therapeutic vulnerabilities for better mitigation of metastatic cancer. The induction of cell death has been the prime target for metastatic cancers for decades. This includes chemo-, immuno-, and radiotherapies. These therapeutics are, of course, efficient in inducing cell death in multiple cancer types in the metastatic cancer context however, the survival after the commencement of the cell death process has to be taken into consideration to prevent relapse. This pivotal reversal of cell death is primarily done by IAPs at the mitochondrial, caspase, and death receptor levels in the contexts of reversible apoptosis, necroptosis, and ferroptosis (Fig. 7). Smac mimetics, in particular, bivalent Smac mimetics stand out to combat reversal of cell death process by inducing robust cell death with death ligands,^19,106,256,257^ chemotherapeutics,^258,259^ and immunotherapeutic agents.^260,261^ However, enhanced toxicity should be considered when a chemotherapeutic agent is combined with Smac mimetics for therapy, as it may elicit tumor lysis syndrome. K-Ras is a game changer in the context of reversal of cell death^19^ as it converts death receptor signaling into metastasis promoting signaling.^252^ Therefore, K-Ras inhibitors could potentially inhibit the reversal of the cell death process during the chemo- or immunotherapeutic-induced cell death process.^21^ The ferroptosis inhibitor GPX4 stands at the crossroads of reversal/inhibition of apoptosis,^262^ necroptosis,^263,264^ and ferroptosis^130,265^ and is transcriptionally promoted specifically during blebbishield-mediated transformed state of cancer stem cells.^93^ RSL3, ML210, ML162, and ALOX inhibitors could target GPX4-directed reversal and or inhibition of multiple cell death processes^266^ (Fig. 7). Despite GPX4 is relevant to drug resistance,^267^ GPX4 targeting should be considered in the context of acute nephrotoxicity.^268^

GPX4 is a prominent link that connects the BMW pathway with the EMT pathway of metastasis as GPX4 is specifically expressed in transformed state spheroids in blebbishield emergency program^93^ and is expressed in the TGF-β-Zeb1-dependent drug-resistant EMT state.^266^ Therefore, Zeb1-GPX4 axis inhibitors described above also serve as prominent EMT inhibitors (comprehensively reviewed with more EMT inhibitors^269^ and the relevant ones to this review are summarized in Fig. 7). Suramin, 1400 W, and iNOS inhibitors (L-NAME and L-NMMA) block EMT-transcription to inhibit EMT^269^ and therefore could be useful in BMW pathway as iNOS plays an essential role in the cell death context^270,271^ (Fig. 7). Rolipram, inhibits phosphodiesterase-4 (PDE4) to inhibit TGF-β induced EMT^269^ and can also promote apoptosis in K-Ras active cells^272^ (Fig. 7). Aldoketoreductase such as AKR1B1 induces EMT in the context of high glucose^273^ and activates GSH-GPX4 axis through glutathione upregulation^274^ suggesting the contribution to multi-cell death resistance to fit in BMW pathway. Targeting AKR1B1 in this context sensitized lung cancer cells to EGFR inhibitors^274^ (Fig. 7). Glycolysis inhibitors such as metformin and 2-deoxyglucose or HMG-CoA inhibition by simvastatin block EMT^269^ as well as promote (secondary) necrosis^275–277^ and thus form an attractive target to block reversal of apoptosis (Fig. 7). Ascorbate, another secondary necrosis inducer by remodeling caspase-8 processing-pattern with antioxidant activity,^260^ is also capable of inhibiting EMT.^269^ Cox-2 inhibition also targets both EMT^269^ and BMW pathways (PIM-1 translation^106^) (Fig. 7). Finally, ROS-induced DNA damage during cell death is in part due to inactivation of PARP1 and cells that retain PARP1 during FasL-induced apoptosis is able to undergo blebbishield emergency program mediated survival.^19^ PARP1 not only helps to repair DNA damage that occurred during apoptosis but also promotes EMT.^269^ Therefore, PARP inhibition is important to target EMT as well as reversal of cell death (Fig. 7). Thus, a therapeutic strategy targeting both EMT and BMW pathways of metastasis is a viable option to combat metastasis.

## Conclusion and future perspectives

In conclusion, metastasis can occur through two overlapping yet distinct routes: the reversible EMT pathway and the BMW pathway. The reversible EMT pathway mainly deals with the change in the cell surface proteome remodeling-coupled to EMT transcription and intermediate filament architecture to adapt to the migratory and invasive phase of metastasis when exhibiting mesenchymal phenotype and switches back towards epithelial phenotype during colonization at preconditioned distant metastatic sites. These plasticity events are driven by metabolic switching plus TGF-β-directed transcription but with suppression of TGF-β’s ability to induce apoptosis. BMW pathway, on the other hand, executes metastasis through the reversible cell death-coupled transformed spheroid state (exhibits resistance to apoptosis, necroptosis, ferroptosis, and possibly other cell death types), cancer cell-to-immune cell fusion, and iDAMP release to acquire immune privilege, migratory capacity from immune cell fusion partner, and colonization through microenvironmental cues that trigger a stop/homing signal for immune cell migration. However, much has to be understood about the BMW pathway of metastasis about the immune cell fusion partners and associated target organ settlement. The major advantage of BMW pathway recognition is that it better explains therapy resistance as the blebbishield emergency program can contribute to rounds and rounds of apoptosis-survival cycles (at least two consecutive cycles were tested and then confirmed its ability form tumors, metastasis, and homing at immune cell-rich organs such as liver and spleen: RT4P-BSE-2 cells^18,93^) resulting in the selection of cells with enhanced drug resistance, boosted glycolysis and augmented chromosomal instability.^19,93^ Furthermore, the knowledge of the BMW pathway gives suggestions on the timing of treatment: for example, when to use or when not to use BMW pathway inhibitors. It is ideal to use a BMW pathway inhibitor when the cell death process and cell death timing is expected (such as therapeutic intervention). More understanding of both EMT and BMW pathways of metastasis will therefore pave the way for better design of therapeutics (chemo and immuno-arms) or educated combination therapeutics to mitigate metastatic cancers in the future.
